# Supplementary material for: Multi-trophic occupancy modeling connects temporal dynamics of woodpeckers and beetle sign following fire
Source: PLoS One. 2023 Mar 6;18(3):e0281687. doi: 10.1371/journal.pone.0281687 (PMC9987826; doi:10.1371/journal.pone.0281687)
Supplement: Attachment — Submitted filename: PONE-D-22-28125_reviewer.pdf [file pone.0281687.s001.pdf]

PLOS ONE

Multi-trophic occupancy modeling connects temporal dynamics of woodpeckers and beetle sign following fire

--Manuscript Draft--

**Manuscript Number:**	PONE-D-22-28125
**Article Type:**	Research Article
**Full Title:**	Multi-trophic occupancy modeling connects temporal dynamics of woodpeckers and beetle sign following fire
**Short Title:**	Connecting woodpecker occurrence to prey sign
**Corresponding Author:**	Morgan W. Tingley<br>University of California Los Angeles<br>Los Angeles, CA UNITED STATES
**Keywords:**	Picoides arcticus; black-backed woodpecker; wildfire; Buprestidae and Cerambycidae; California; insect abundance
**Abstract:**	In conifer forests of western North America, wildlife populations can change rapidly in the decade following wildfire as trees die and animals respond to concomitant resource pulses that occur across multiple trophic levels. In particular, black-backed woodpeckers (Picoides arcticus) show predictable temporal increases then declines following fire that have been hypothesized to be a response to their main prey: woodboring beetle larvae of the families Buprestidae and Cerambycidae. Here, we pair woodpecker surveys over 10 years with surveys of woodboring beetle sign and activity, collected at 128 survey plots across 22 recent fires, to ask whether accumulated beetle sign indicates current or past black-backed woodpecker occurrence, and whether that relationship is mediated by the number of years since fire. We test this relationship using an integrative multi-trophic occupancy model. Our results demonstrate that woodboring beetle sign is a positive indicator of woodpecker presence 1–3 years following fire, an uninformative indicator from 4-6 years after fire, and a negative indicator beginning 7 years following fire. Woodboring beetle activity, itself, is temporally variable and dependent on tree species composition, with beetle sign generally accumulating over time, particularly in stands with diverse tree communities, but decreasing over time in Pinus-dominated stands where faster bark decay rates lead to brief pulses of beetle activity followed by rapid degradation of tree substrate and accumulated beetle sign. Altogether, the strong connections of woodpecker occurrence to beetle activity support prior hypotheses of how multi-trophic interactions govern rapid temporal dynamics of primary and secondary consumers in burned forests. While our results indicate that beetle sign is, at best, a rapidly shifting and potentially misleading measure of woodpecker occurrence, the better we understand the interacting mechanisms underlying temporally dynamic systems, the more successfully we will be able to predict the outcomes of management actions.
**Order of Authors:**	Morgan W. Tingley
	Graham A. Montgomery
	Robert L. Wilkerson
	Daniel R. Cluck
	Sarah C. Sawyer
	Rodney B. Siegel
**Additional Information:**	
**Question**	**Response**
**Financial Disclosure**<br><br>Enter a financial disclosure statement that describes the sources of funding for the	Funding for analysis and research came from the USDA Forest Service. The funders had no role in study design, data collection and analysis, decision to publish, or preparation of the manuscript.

Powered by Editorial Manager® and ProduXion Manager® from Aries Systems Corporation

work included in this submission. Review the submission guidelines for detailed requirements. View published research articles from *PLOS ONE* for specific examples.<br><br>This statement is required for submission and **will appear in the published article** if the submission is accepted. Please make sure it is accurate.<br><br>**Unfunded studies**<br>Enter: *The author(s) received no specific funding for this work.*<br><br>**Funded studies**<br>Enter a statement with the following details:<br>• Initials of the authors who received each award<br>• Grant numbers awarded to each author<br>• The full name of each funder<br>• URL of each funder website<br>• Did the sponsors or funders play any role in the study design, data collection and analysis, decision to publish, or preparation of the manuscript?<br>• **NO** - Include this sentence at the end of your statement: *The funders had no role in study design, data collection and analysis, decision to publish, or preparation of the manuscript.*<br>• **YES** - Specify the role(s) played.<br><br>* typeset	
**Competing Interests**<br><br>Use the instructions below to enter a competing interest statement for this submission. On behalf of all authors, disclose any competing interests that could be perceived to bias this work—acknowledging all financial support and any other relevant financial or non-financial competing interests.<br><br>This statement is required for submission and **will appear in the published article** if the submission is accepted. Please make sure it is accurate and that any funding sources listed in your Funding Information	The authors have declared that no competing interests exist.

later in the submission form are also declared in your Financial Disclosure statement.<br><br>View published research articles from *PLOS ONE* for specific examples.<br><br>**NO authors have competing interests**<br><br>Enter: *The authors have declared that no competing interests exist.*<br><br>**Authors with competing interests**<br><br>Enter competing interest details beginning with this statement:<br><br>*I have read the journal's policy and the authors of this manuscript have the following competing interests: [insert competing interests here]*<br><br>* typeset	
**Ethics Statement**<br><br>Enter an ethics statement for this submission. This statement is required if the study involved:<br><br>• Human participants<br>• Human specimens or tissue<br>• Vertebrate animals or cephalopods<br>• Vertebrate embryos or tissues<br>• Field research<br><br>Write "N/A" if the submission does not require an ethics statement.<br><br>General guidance is provided below. Consult the submission guidelines for detailed instructions. **Make sure that all information entered here is included in the Methods section of the manuscript.**	N/A

**Format for specific study types**<br><br>**Human Subject Research (involving human participants and/or tissue)**<br>• Give the name of the institutional review board or ethics committee that approved the study<br>• Include the approval number and/or a statement indicating approval of this research<br>• Indicate the form of consent obtained (written/oral) or the reason that consent was not obtained (e.g. the data were analyzed anonymously)<br><br>**Animal Research (involving vertebrate animals, embryos or tissues)**<br>• Provide the name of the Institutional Animal Care and Use Committee (IACUC) or other relevant ethics board that reviewed the study protocol, and indicate whether they approved this research or granted a formal waiver of ethical approval<br>• Include an approval number if one was obtained<br>• If the study involved *non-human primates*, add *additional details* about animal welfare and steps taken to ameliorate suffering<br>• If anesthesia, euthanasia, or any kind of animal sacrifice is part of the study, include briefly which substances and/or methods were applied<br><br>**Field Research**<br><br>Include the following details if this study involves the collection of plant, animal, or other materials from a natural setting:<br>• Field permit number<br>• Name of the institution or relevant body that granted permission	
**Data Availability**<br><br>Authors are required to make all data underlying the findings described fully available, without restriction, and from the time of publication. PLOS allows rare exceptions to address legal and ethical concerns. See the PLOS Data Policy and FAQ for detailed information.	Yes - all data are fully available without restriction

A Data Availability Statement describing where the data can be found is required at submission. Your answers to this question constitute the Data Availability Statement and **will be published in the article**, if accepted.<br><br>**Important:** Stating 'data available on request from the author' is not sufficient. If your data are only available upon request, select 'No' for the first question and explain your exceptional situation in the text box.<br><br>Do the authors confirm that all data underlying the findings described in their manuscript are fully available without restriction?	
**Describe where the data may be found in full sentences. If you are copying our sample text, replace any instances of XXX with the appropriate details.**<br><br>• If the data are **held or will be held in a public repository**, include URLs, accession numbers or DOIs. If this information will only be available after acceptance, indicate this by ticking the box below. For example: *All XXX files are available from the XXX database (accession number(s) XXX, XXX.).*<br>• If the data are all contained **within the manuscript and/or Supporting Information files**, enter the following: *All relevant data are within the manuscript and its Supporting Information files.*<br>• If neither of these applies but you are able to provide **details of access elsewhere**, with or without limitations, please do so. For example:<br><br>*Data cannot be shared publicly because of [XXX]. Data are available from the XXX Institutional Data Access / Ethics Committee (contact via XXX) for researchers who meet the criteria for access to confidential data.*<br><br>*The data underlying the results presented in the study are available from (include the name of the third party*	Input data and model code in JAGS language are available from the Dryad Digital Repository <URL to be filled in upon acceptance> (Tingley et al., XXXX).

and contact information or URL).<br>• This text is appropriate if the data are owned by a third party and authors do not have permission to share the data.<br><br>* typeset	
Additional data availability information:	Tick here if the URLs/accession numbers/DOIs will be available only after acceptance of the manuscript for publication so that we can ensure their inclusion before publication.

Click here to access/download;Manuscript;MainText_PLOSONE.docx

Multi-trophic occupancy modeling connects temporal dynamics of woodpeckers and beetle sign following fire

Running title: Connecting woodpecker occurrence to prey sign

Morgan W. Tingley[1*], Graham A. Montgomery[1], Robert L. Wilkerson[2], Daniel R. Cluck[3], Sarah C. Sawyer[4], and Rodney B. Siegel[2]

[1]Ecology and Evolutionary Biology, University of California, Los Angeles, CA, USA

[2]The Institute for Bird Populations, Petaluma, CA, USA

[3]USDA Forest Service, Forest Health Protection, Susanville, CA, USA

[4]USDA Forest Service, Pacific Southwest Region, Vallejo, CA, USA

* Corresponding author:

Email: mtingley@ucla.edu (MWT)

ABSTRACT

In conifer forests of western North America, wildlife populations can change rapidly in the decade following wildfire as trees die and animals respond to concomitant resource pulses that occur across multiple trophic levels. In particular, black-backed woodpeckers (*Picoides arcticus*) show predictable temporal increases then declines following fire that have been hypothesized to be a response to their main prey: woodboring beetle larvae of the families Buprestidae and Cerambycidae. Here, we pair woodpecker surveys over 10 years with surveys of woodboring beetle sign and activity, collected at 128 survey plots across 22 recent fires, to ask whether accumulated beetle sign indicates current or past black-backed woodpecker occurrence, and whether that relationship is mediated by the number of years since fire. We test this relationship using an integrative multi-trophic occupancy model. Our results demonstrate that woodboring beetle sign is a positive indicator of woodpecker presence 1–3 years following fire, an uninformative indicator from 4-6 years after fire, and a negative indicator beginning 7 years following fire. Woodboring beetle activity, itself, is temporally variable and dependent on tree species composition, with beetle sign generally accumulating over time, particularly in stands with diverse tree communities, but decreasing over time in *Pinus*-dominated stands where faster bark decay rates lead to brief pulses of beetle activity followed by rapid degradation of tree substrate and accumulated beetle sign. Altogether, the strong connections of woodpecker occurrence to beetle activity support prior hypotheses of how multi-trophic interactions govern rapid temporal dynamics of primary and secondary consumers in burned forests. While our results indicate that beetle sign is, at best, a rapidly shifting and potentially misleading measure of woodpecker occurrence, the better we understand the interacting mechanisms underlying

temporally dynamic systems, the more successfully we will be able to predict the outcomes of management actions.

Keywords: *Picoides arcticus*; black-backed woodpecker; wildfire; Buprestidae and Cerambycidae; California; insect abundance

INTRODUCTION

Dead and dying trees are the key resource driving post-fire biodiversity pulses in the western United States, as fire unlocks hundreds of years of stored energy in the form of cellulose and other plant tissue [1]. Fire-killed trees attract insects – particularly woodboring beetles of the families Cerambycidae and Buprestidae – which lay their eggs on or in the bark of dead trees. For many years after a fire, woodboring beetle larvae feed on the accessible wood tissue, forming "galleries" underneath the bark [2]. Beetle larvae are in turn a desirable food resource, and their presence attracts woodpeckers of multiple species, which nest and forage in high densities in recent post-fire forests [3,4]. The nest holes of woodpeckers subsequently attract and shelter many other wildlife species as well, including owls, flying squirrels, and a variety of secondary cavity-nesting passerine birds [5].

Food resource availability can predict abundance or occurrence of predators, but only if food resource availability is a primary limiting factor of population size [6,7]. Different bird populations show evidence both for [8–10] and against [11] population limitation due to specific resource availability, and knowledge of the factors limiting species presence can be important for conservation and management. This is especially true for species of conservation concern like the black-backed woodpecker (*Picoides arcticus*), a species which is closely associated with post-fire conifer forests in western North America. Black-backed woodpeckers are known to forage primarily on the larvae of woodboring beetles [4], although recent dietary analysis indicates a wider dietary breadth that commonly includes insects of the orders Diptera, Araneae, and Hymenoptera [12]. Nevertheless, activity and abundance of woodboring beetles declines with time since fire [13], mirroring a temporal pattern of occupancy and abundance that has also been well-documented in black-backed woodpeckers [14,15], and suggesting that woodboring

beetle larvae abundance may fundamentally be driving the post-fire dynamics of black-backed woodpeckers.

Finding further similarities in spatial and temporal patterns of both woodboring beetles and black-backed woodpeckers could further clarify the degree of linkage between black-backed woodpecker population size and beetle prey abundance. Links between black-backed woodpeckers and their beetle prey would also suggest that monitoring for one may provide inference on the abundance of the other. If a strong link exists, it could enable more efficient assessment of cross-trophic, post-fire biodiversity from relatively simple, standard surveys. For example, beetle surveys could be used as a proxy for woodpecker abundance in some cases.

Post-fire temporal dynamics of both woodpeckers and woodboring beetles could also depend on forest composition. Ray et al. (2019) documented that beetle activity varied by tree species, with trees of genus *Pinus* decaying more quickly and thus harboring larger numbers of woodboring beetles in the first few years following fire. Dominant tree species has been hypothesized to impact occurrence patterns of black-backed woodpeckers [14], but a mechanistic relationship tying woodpeckers to particular tree species as a function of time since fire has never been clarified.

In 2018, we surveyed for both larval woodboring beetles and black-backed woodpeckers at 128 points arrayed across 22 fires that burned sometime in the previous ten years. Using beetle larvae sign to create a cumulative index of ‘food availability’, we ask the following two questions: 1) do food resources predict black-backed woodpecker occurrence? and 2) do different tree species differentially provide foraging resources for black-backed woodpeckers in burned forests? We provide a novel multi-trophic occupancy modeling framework to answer

these questions and connect tree species, beetle larvae surveys, and woodpecker detections across burned forests throughout the decade after fire.

METHODS

Study area and survey methods

We conducted black-backed woodpeckers surveys as part of a long-term project to monitor bird occupancy and trends following forest fire in montane forests of eastern California. Our study area comprised ten contiguous National Forest units within the Sierra Nevada and Southern Cascades ecoregions of California (Fig. 1), with forest types dominated by Sierra mixed conifer (primarily *Pinus ponderosa, P. lambertiana*, *Abies concolor*, *Pseudotsuga menziesii*, *Calocedrus decurrens*, and *Quercus kelloggii*) and eastside pine (*Pinus ponderosa* dominated with some *P. jeffreyi*). Higher elevation areas contain larger proportions of firs (*A. concolor and A. magnifica*) and *Pinus contorta*. In this study region, we randomly selected 50 fires to visit in 2018 that met our sampling criteria of having burned within the previous 10 years and containing at least 50 ha of conifer forest that burned at mid- or high-severity in one of the ten target National Forest units. Many of these fires had been surveyed for black-backed woodpeckers one or more times during the preceding decade, yielding data which we also used in this study. At each of the 50 fires, we randomly generated a target starting location within the fire perimeter, and crews established black-backed woodpecker survey transects beginning as close as possible to the target location. Each transect consisted of approximately 20 survey points, located a minimum of 250 m from each other to minimize double-counting of individuals. All surveys included a 6-minute broadcast survey (subdivided into three 2-minute detection intervals), during which electronic broadcasts of black-backed woodpecker vocalizations and territorial drumming

(obtained from The Macaulay Library of Natural Sounds, Cornell Laboratory of Ornithology; recorded by G.A. Keller) were played for 30 seconds, followed by a 1.5-minute silent observation period. At alternating points, broadcast surveys were preceded by a 7-minute passive survey (subdivided into detection intervals of 3, 2, and 2 minutes, respectively). We followed a removal methodology where call broadcasts were suspended after the first detection. We conducted surveys in the morning hours (0530–0930) between 4 May and 18 July each year.

Fig. 1. Locations of the 22 fires (red dots) surveyed for woodboring beetles and black-backed Woodpeckers within U.S. National Forest units in California.

In 2018, we also conducted targeted woodboring beetle surveys at a subset of 22 of the 50 fires visited for black-backed woodpecker surveys. We used preliminary results from our 2018 black-backed woodpecker surveys to select equal numbers of fires to survey for woodboring beetles in each of 3 categories: fires where we detected black-backed woodpeckers at > 50% of survey points; fires where we detected black-backed woodpeckers at < 50% of points; and fires where we detected no black-backed woodpeckers. At each fire we randomly selected six woodpecker survey points at which to conduct woodboring beetle surveys. Up to three of those points were drawn from points where Black-backed Woodpeckers had been detected earlier that summer; the remaining points were drawn from the points at which black-backed woodpeckers were not detected that year.

Woodboring beetle surveys involved assessing the six closest snags to each selected black-backed woodpecker survey point for larvae activity and sign, and overall condition. Larvae activity and sign were assessed on and under each of two 15 cm x 15 cm bark samples that were

removed from the tree, one from the north side of the trunk and one from the south side of the trunk, taken at the DBH line. Each bark sample was given a single, holistic woodboring beetle activity score determined by presence/absence of activity by sample quadrant on bark exterior, interior and sapwood (frass/boring dust, exit/entrance holes, galleries, and presence of larvae). Tree species and physical characteristics (DBH, tree height, high/low bark char heights, needle presence and color) were collected at each snag. Detailed data collection procedures are provided in Ray et al. (2019).

Our analysis dataset consisted of surveys for black-backed woodpeckers and beetle activity at 128 points located within 22 fires (Table 1). While beetle surveys were conducted only in 2018, fires ranged in their number of years since fire (1–10) and in the number of years they had been surveyed previously for black-backed woodpeckers prior to 2018 (28 points surveyed in 0 prior years, 24 points in 1 year, 6 points in 2 years, 24 points in 3 years, 6 points in 4 years, 12 points in 5 years, 11 points in 6 years, 12 points in 7 years, and 5 points in 8 years). We used woodpecker survey data from all previous visits – in addition to our 2018 woodpecker and beetle surveys – to model changing woodpecker occurrence over time as a function of beetle sign evident in 2018.

Table 1. Fires surveyed for both black-backed woodpeckers and woodboring beetles. Beetle surveys were conducted in 2018 and woodpecker surveys were conducted in 2018 and up to 8 additional years prior.

Fire Name	Year Burned	U.S. National Forest Unit	No. of Beetle Survey Points	Black-backed Woodpecker Survey Years
Aspen	2013	Sierra	6	2014, 2015, 2016, 2017, 2018
Bald	2014	Lassen	6	2015, 2016, 2017, 2018

Barry Point	2012	Modoc	6	2013, 2014, 2015, 2018
Clark	2016	Inyo	6	2017, 2018
Cold	2008	Plumas	6	2012, 2013, 2014, 2016, 2017, 2018
Cougar	2011	Modoc	6	2012, 2014, 2015, 2018
Cove	2017	Modoc	6	2018
Fox	2008	Plumas	6	2011, 2013, 2014, 2015, 2016, 2017, 2018
Frog	2015	Modoc	6	2016, 2017, 2018
George	2012	Sequoia	5	2013, 2014, 2015, 2016, 2017, 2018
Granite	2009	Sequoia	6	2010, 2011, 2013, 2014, 2015, 2016, 2017, 2018
Lion	2009	Sequoia	6	2010, 2011, 2014, 2013, 2015, 2016, 2017, 2018
Minerva 5	2017	Plumas	6	2018
Onion 2	2008	Lassen	5	2010, 2011, 2012, 2013, 2014, 2015, 2016, 2017, 2018
Owens River	2016	Inyo	6	2017, 2018
Peak	2012	Plumas	6	2015, 2016, 2017, 2018
Pier	2017	Sequoia	6	2018
Railroad	2017	Sierra	6	2018
Rough	2015	Sierra	4	2017, 2018
Scotch	2008	Plumas	6	2009, 2010, 2012, 2013, 2014, 2015, 2018
Soup 2	2016	Modoc	6	2017, 2018
Steele	2017	Modoc	6	2018

Analytical Approach

We developed a hierarchical model in a Bayesian context to jointly model both the dynamics of beetle activity intensity over time within our plots, as well as the occurrence – accounting for imperfect detection – of black-backed woodpeckers at those same plots. The model largely follows the structure of a single-species occupancy model [16], where woodpecker observations of detection or non-detection, y_{jkt}, for survey interval *k* at site *j* (where sites are individual survey points) in year *t*, are assumed to be imperfectly observed representations of the true occurrence status, z_{jt} (present or absent), which is constant across all *k* survey intervals (i.e., closure is assumed within the <17-minute survey period) but can change from year to year. Observed occurrence of black-backed woodpeckers, y_{jkt}, is thus modeled as

$$y_{jkt} \sim \text{Bernoulli}(z_{jt} p_{jkt}), \quad (1)$$

where p_{jkt} is the probability of detection for a given survey at a site. Similarly, the true occurrence status of a site in year *t*, z_{jt}, is modeled as

$$z_{jt} \sim \text{Bernoulli}(\psi_{jt}), \quad (2)$$

where ψ_{jt} is the probability of occurrence at a site.

The probabilities of woodpecker detection and occurrence are both modeled as logit-linear functions of *a priori* hypothesized covariates. Following previous work studying black-backed woodpeckers with this survey methodology [14,15,17], we expected detection, p_{jkt}, to vary as a function of an intercept and the linear additive combination of a categorical covariate representing the survey type (passive = 0, broadcast = 1), giving

$$\text{logit}(p_{jkt}) = \alpha_0 + \alpha_{type}\text{type}_k. \quad (3)$$

The probability of woodpecker occupancy of a survey point was modeled as a function of six covariates: (1) elevation, (2) latitude, (3) snag density, (4) intensity of beetle larvae activity

(as indirectly measured by cumulative beetle sign since the fire; modeled as a latent variable, see below), and (5) an interaction between years-since-fire and the intensity of beetle larvae activity (with the hypothesis that cumulative beetle sign becomes less predictive over time). Snag counts were conducted immediately after completing woodpecker surveys and consisted of counting all snags of different size classes (10-30, 30-60, and >60 cm dbh) within 50 m of each survey point. Size-specific snag counts were aggregated in the field into different categories (≤5, 6-15, 16-30, 31-50, 51-100, >100), which were converted to numerical quantities (1, 6, 16, 31, 51, 101, respectively) for analysis [15]. Counts across all three size classes were summed in order to calculate snag density (snags/ha). The linear additive model for occupancy in the first year of surveys can be described as

$$\text{logit}(\psi_{j,t=1}) = \beta_0 + \beta_{elev}\text{elev}_j + \beta_{lat}\text{lat}_j + \beta_{snag}\text{snag}_{jt} + \beta_{beetle}\text{intensity}_{jt} + \beta_{ageXbeetle}\text{age}_{jt}\text{intensity}_{jt}, \quad (4)$$

where β represents intercept and slope parameters. To account for pseudoreplication and temporal autocorrelation derived by sampling sites repeatedly in consecutive years, we added a temporal autocorrelation term [18], ϕ, which was multiplied by the true occurrence status in year *t-1*, resulting in the following model for additional post-fire years,

$$\text{logit}(\psi_{j,t>1}) = \beta_0 + \beta_{elev}\text{elev}_j + \beta_{lat}\text{lat}_j + \beta_{snag}\text{snag}_{jt} + \beta_{beetle}\text{intensity}_{jt} + \beta_{ageXbeetle}\text{age}_{jt}\text{intensity}_{jt} + \phi z_{j,t-1} \quad . \quad (5)$$

As 2018 was the last year of surveys used in this dataset, and also the only year with *in situ* beetle activity surveys, all surveys conducted in 2018 held the temporal index of t=10. Surveys in previous years (t = 1,…,9) were treated as missing data if no surveys occurred at a site in that survey-year.

A novel feature of our multi-trophic model is that we treat cumulative beetle larvae sign at a survey point in a given year (intensity_{jt}) as a latent (i.e., indirectly observed), continuous variable. We are then able to model beetle larvae sign as a function of different environmental variables hypothesized to relate to beetle activity and to account for the known dynamic that beetle sign generally accumulates over time even though overall activity may decline. Thus, we hypothesized that the intensity of beetle sign at a site in a given year (intensity_{jt}) varies as a function of: (1) the number of years since fire; (2) the proportion of sampled trees per point that were of the genus *Pinus*; (3) and an interaction between the proportion of pines and years since fire. Based on previous work [13], we hypothesized that beetle sign increases over time (as sign is generally cumulative and lasting), but that pines would have greater activity in early post-fire years and lower activity in later post-fire years (as pine bark generally decomposes faster than bark of other trees in our study areas). We thus modeled beetle sign intensity as,

$$\text{logit}\left(intensity_{jt}\right) = \gamma_0 + \gamma_{age}\text{age}_{jt} + \gamma_{pine}\text{pine}_j + \gamma_{ageXpine}\text{age}_{jt}\text{pine}_j. \quad (6)$$

We fit this model to observed data collected in 2018, by treating the total sum of beetle sign scores across all surveyed trees per point (max = 6) as a binomially distributed variable, as follows,

$$\text{activity}_j \sim binomial(intensity_{j,t=10}, \text{numTrees}_j * 8), \quad (7)$$

where the maximum activity score is a product of the number of trees sampled per point (numTrees_j) and the maximum potential activity score per tree (i.e., 8).

We fit the model to the data with JAGS [19] using the R statistical programming language version 4.0.2 (R Core Team, 2019) and the package ‘R2jags’ [21]. We used vague priors (i.e., normal with μ = 0, τ = 0.1). We ran three chains of 50,000 iterations thinned by 50 with a burn-in of 50,000, yielding a posterior sample of 3,000 across all chains. Convergence

was checked visually with traceplots and confirmed with a Gelman-Rubin statistic < 1.1 [22] . Inference on parameters was made using 95% Bayesian credible intervals (95 CI).

RESULTS

Do food resources predict recent black-backed woodpecker occurrence?

We found no temporally consistent relationship between woodpecker occupancy and the intensity of beetle sign (β_{beetle} = 0.74, 95% CI = -2.33, 3.88), but a strong interactive relationship of beetle sign intensity with fire age on woodpecker occupancy ($\beta_{ageXbeetle}$ = -0.88, 95% CI = -1.38, -0.40). This strong relationship indicates that in the first few years following fire, beetle sign is a positive indicator of black-backed woodpecker occupancy, but by 10 years after fire, beetle sign is a negative indicator of black-backed woodpecker occupancy (Fig. 2).

Fig. 2. Black-backed woodpecker occupancy as a function of an index of intensity of woodboring beetle sign, with the relationship differing depending on the number of years since fire (only 5 years shown, for clarity). Solid lines represent posterior means and ribbons represent partial 95% credible intervals representing uncertainty only in the interaction between beetle sign and years since fire.

Do different tree species differentially provide foraging resources for black-backed woodpeckers over time in post-fire forests?

Consistent with our hypotheses, beetle sign was higher in plots with a greater proportion of pine trees (γ_{pine} = 0.36, 95% CI = 0.20, 0.53) and also generally increased over time following fire (γ_{age} = 0.30, 95% CI = 0.20, 0.32). However, we found a strong negative interaction between

these two variables on beetle sign intensity ($\gamma_{\mathrm{ageXpine}}$ = -0.62, 95% CI = -0.74, -0.49), such that beetle sign increases over time in plots that are primarily trees other than of the genus *Pinus*, but actually decreases over time when plots are mostly pine trees (Fig. 3).

Fig. 3. Index of intensity of woodboring beetle sign as a function of the number of years post-fire and the percentage of trees of the genus *Pinus* sampled in each plot. Solid lines represent posterior means and ribbons represent 95% credible intervals around the predicted intensity index.

Additional findings

We found positive associations of occupancy with higher elevation and latitude, a weak positive association of occupancy with snag density (controlling for beetle activity), and a strong effect of survey type (passive vs. broadcast surveys) on detectability (Table 2).

Table 2. Posterior estimates of model parameters. Slope parameters with 95% Bayesian credible i[illegible]ervals that do not cross zero suggest strong relationships and are highlighted in bold.

Response	Parameter	Estimate	Lower 95% CI	Upper 95% CI
Woodpecker detection	Intercept	-5.16	-7.19	-3.74
-	**Survey type**	5.43	3.93	7.51
Woodpecker occupancy	Intercept	-1.59	-3.18	-0.08
-	Beetle sig[illegible]	0.74	-2.33	3.88
-	**Beetle sign * fire age**	-0.88	-1.38	-0.40
-	**Elevation**	1.19	0.78	1.69
-	**Latitude**	0.98	0.60	1.46
-	Snag density	0.15	-0.01	0.31
-	**Temporal autocorrelation**	0.94	0.16	1.74
Beetle activity	Intercept	-0.13	-0.22	-0.04
-	**Proportion of pines**	0.36	0.19	0.54
-	**Years since fire (fire age)**	0.26	0.20	0.32
-	**Proportion of pines * fire age**	-0.62	-0.74	-0.49

DISCUSSION

We found strong relationships between woodpecker occurrence and the intensity of beetle sign; however, the magnitude and direction of these relationships changes rapidly over time. In particular, beetle sign 1–3 years post-fire appears to be a good indicator of occupancy by black-backed woodpeckers during that time period – perhaps even a better indicator than snag density (which is often used as a spatial indicator of woodpecker occupancy within fires; [14,17,23].

However, between 4–7 years post-fire, the signal from beetle sign becomes muddled, with little to no relationship between beetle sign and woodpecker occupancy. By 10-years post-fire, the relationship has reversed, with areas of high beetle sign showing lower black-backed woodpecker occupancy.

The likely reason behind this inverse relationship is that beetle activity sign should monotonically accumulate over time in the first decade following fire (until trees decay so much that sign deteriorates), even if beetle activity, itself, strongly declines with time [24,25]. For example, a particular tree may only contain large numbers of beetle larvae for the first 3–4 years following fire, but may continue to accumulate beetle activity sign gradually for a full decade. Consequently, by 10 years post-fire, the accumulated sign of woodboring beetles may be a better indicator of past – rather than present – black-backed woodpecker occurrence.

The expected pattern of accumulation of beetle sign is compounded by temporal dynamics of beetle activity and wood decay that differ by tree species. In the middle elevations of the Sierra Nevada, where the majority of canopy conifers are either of the genus *Pinus* or *Abies* (with patches of *Pseudotsuga* and *Calocedrus*), woodboring beetle temporal dynamics can differ markedly by tree species [13], as different tree genera decay and fall at different rates, while also subject to variation owing to tree size and local conditions [26–28]. Trees of the genus *Pinus* generally have vascular tissue that quickly becomes suitable for feeding larvae following mortality, leading to rapid post-fire colonization by woodboring beetles [27]. As part of the decay process, pine bark sloughs off relatively quickly, as the vascular tissue dries out or is completely consumed by beetle larvae. This leaves a hardened tree bole exposed to the elements and unsuitable for larvae, vastly reducing the role of *Pinus* trees as beetle larvae reservoirs several years after death. Vascular tissue of other trees such as those of the genus *Abies*, by

comparison, does not become suitable for woodboring beetles as quickly following death [27] – possibly due to different under-bark microclimates or differences in host volatile attraction [13] – so plots with primarily non-*Pinus* trees show patterns of beetle intensity that increase over time, rather than decrease (Fig. 3). The temporal difference in beetle suitability between *Pinus* and *Abies* trees may be further exacerbated by the long-term fate of snags of each. *Pinus* snags tend to fall over after death – possibly due to destabilization by a woodboring beetle that targets pine roots interacting with appropriate decay microclimate and fungi [28] – which could limit beetle colonization and woodpecker foraging, while *Abies* snags tend to snap half-way or near the crown, providing longer-term foraging resources [29–32].

As a methodological note, we were able to uncover these relationships due to the development of a novel multi-trophic occupancy model. Multi-species occupancy models primarily either treat multiple detected species as occurring independently in a random-effects framework [33,34] or directly interacting with the occupancy and/or detectability of other species in frameworks meant to model just a handful of species [35,36]. In both cases, however, models generally assume that all included species are detected or surveyed through the same type of method and at identical spatial and temporal sampling scales. In our system, woodpecker occurrence (measured via point counts) is potentially influenced by woodboring beetle abundance, which is assumed to be unaffected by woodpecker occurrence; but critically, woodboring beetle abundance is indirectly assessed via accumulating beetle sign from up to six trees at each woodpecker survey point. Thus, our model integrates survey data on two trophic levels [37] within a hierarchical system where abundance of the lower trophic level potentially impacts the occurrence of the higher trophic level. Such a model structure could be easily

generalized to other multi-trophic systems, where survey assessment of each trophic level generally follows different methodologies each with its own unique observation process.

Conclusions

Rapid and reliable survey methodologies are critical for land managers tasked with making decisions following fire across large land expanses. While systematic bird surveys (e.g. point counts) are well established monitoring tools, woodboring beetle surveys in post-fire areas have held an intriguing allure due to their potential to provide multi-trophic inference on both insects and their predators (e.g., woodpeckers). Our beetle survey methods – which integrated assessments of frass, boring dust, exit and entrance holes, galleries, and direct counts of larvae – are comprehensive yet complicated by the cumulative nature of nearly all of these forms of beetle sign (larvae counts being the exception). This accumulation of sign interacts with the decomposition and decline of the structural integrity of snags, which ultimately leads to a complex and non-linear relationship of beetle sign with woodpecker occurrence (Fig. 2). Ultimately, based on these results, beetle sign in general should not be used as a proxy for woodpecker abundance after 2 or 3 years following fire, and may also not be a good proxy for beetle abundance after that point (although we did not directly assess beetle abundance). In the initial 1–2 years following fire, when rapid management decision-making is often most critical, however, beetle sign may be a reliable method for assessing immediate multi-trophic responses to post-fire conditions. Nevertheless, given the non-linear temporal dynamics of black-backed woodpeckers in post-fire forests [15], combined with the apparent shifting usage of tree species with time since fire both for woodboring beetles and their predators (i.e., from *Pinus* to *Abies*), the portions of burned forests that harbor high beetle and/or black-backed woodpecker

abundances immediately after fire may not sufficiently sustain black-backed woodpecker populations over the longer term. Together, these results imply that management activities for black-backed woodpeckers should account not only for where woodpeckers are when post-fire forest management actions are implemented (i.e., usually within 1–3 years following fire), but also consider, as feasible, where the woodpeckers are likely to go in the near future given a shifting mosaic of tree mortality and prey availability.

The nuanced but strong relationshi[illegible]tween woodpecker occurrence and beetle activity sign ultimately corroborates that black-backed woodpecker populations in western forests depend heavily on woodboring beetle abundan[illegible] a keystone consideration for the management and conservation of black-backed woodpeckers. While this is perhaps an unsurprising finding given prior work in this system and species [1,17,24,38,39], its confirmation is not trivial, as many bird species do not show such prey-dependence [7] and black-backed woodpecker diets show a great diversity of insect prey [12]. While many previous studies have shown strong positive relationships between black-backed woodpeckers and availability of dead trees [14,17,39–42], we found no effect of snag density on woodpecker occupancy while simultaneously accounting for beetle sign (Table 2). Snag density has long been considered a proxy for food availability in this system [42], even though snags are also used predominantly for nesting [43,44]. Our results thus confirm what has long been indirectly hypothesized about black-backed woodpeckers, that their fine-scale spatial distribution is strongly impacted by the spatiotemporal dynamics of prey availability, which itself varies non-linearly over time following fire and as a function of forest tree composition.

Acknowledgements

We are grateful to the numerous field technicians who have gathered woodpecker occurrence data over many years as part of the Management Indicator Species program, funded by the Pacific Southwest Region of the USDA Forest Service. Funding for analysis and research came from the USDA Forest Service. This is Contribution No. XXX of The Institute for Bird Populations.

Author Contributions

MT, BW, RS, DC, and SS conceived the ideas and designed methodology; BW, RS, and DK oversaw data collection; MT analyzed the data; MT and GM led the writing of the manuscript. All authors contributed critically to the drafts and gave final approval for publication.

Data Accessibility

Input data and model code in JAGS language are available from the Dryad Digital Repository <URL to be filled in upon acceptance> (Tingley et al., XXXX).

References

1. Hutto RL. The ecological importance of severe wildfires: some like it hot. Ecol Appl. 2008;18: 1827–1834.

2. Furniss RL, Carolin VM. Western forest insects. Miscellaneous Publication No. 1339. Washington, D.C.: USDA Forest Service; 1977. Available: https://www.biodiversitylibrary.org/bibliography/131875

3. Villard P, Beninger CW. Foraging behavior of male Black-backed and Hairy Woodpeckers in a forest burn. J Field Ornithol. 1993;64: 71–76.

4. Murphy EC, Lehnhausen WA. Density and foraging ecology of woodpeckers following a stand-replacement fire. Journal of Wildlife Management. 1998;62: 1359–1372.

5. Trzcinski MK, Cockle KL, Norris AR, Edworthy M, Wiebe KL, Martin K. Woodpeckers and other excavators maintain the diversity of cavity- nesting vertebrates. J Anim Ecol. 2021. doi:10.1111/1365-2656.13626

6. White TCR. The role of food, weather and climate in limiting the abundance of animals. Biol Rev. 2008;83: 227–248. doi:10.1111/j.1469-185x.2008.00041.x

7. Newton I. The Role of Food in Limiting Bird Numbers. Ardea. 1980;55: 11–30. doi:10.5253/arde.v68.p11

8. Derbyshire R, Strickland D, Norris DR. Experimental evidence and 43 years of monitoring data show that food limits reproduction in a food-caching passerine. Ecology. 2015;96: 3005–3015. doi:10.1890/15-0191.1

9. Nagy LR, Holmes RT. Food limits annual fecundity of a migratory songbird: An experimental study. Ecology. 2005;86: 675–681. doi:10.1890/04-0155

10. Danner RM, Greenberg R, Danner J, Kirkpatrick LT, Walters JR. Experimental support for food limitation of a short-distance migratory bird wintering in the temperate zone. Ecology. 2013; 130610134856005. doi:10.1890/13-0337.1

11. Stephens PA, Vieira MV, Willis SG, Carbone C. The limits to population density in birds and mammals. Storch D, editor. Ecol Lett. 2019;22: 654–663. doi:10.1111/ele.13227

12. Stillman AN, Caiafa MV, Lorenz TJ, Jusino MA, Tingley MW. DNA metabarcoding reveals broad woodpecker diets in fire-maintained forests. Ornithol. 2022;139. doi:10.1093/ornithology/ukac009

13. Ray C, Cluck DR, Wilkerson RL, Siegel RB, White AM, Tarbill GL, et al. Patterns of woodboring beetle activity following fires and bark beetle outbreaks in montane forests of California, USA. Fire Ecol. 2019;15: 21. doi:10.1186/s42408-019-0040-1

14. Saracco JF, Siegel RB, Wilkerson RL. Occupancy modeling of Black- backed Woodpeckers on burned Sierra Nevada forests. Ecosphere. 2011;2: 1–17. doi:10.1890/es10-00132.1

15. Tingley MW, Stillman AN, Wilkerson RL, Howell CA, Sawyer SC, Siegel RB. Cross- scale occupancy dynamics of a postfire specialist in response to variation across a fire regime. Parr C, editor. Journal of Animal Ecology. 2018;209: 333. doi:10.1111/1365-2656.12851

16. MacKenzie DI, Nichols JD, Lachman GB, Droege S, Royle JA, Langtimm CA. ESTIMATING SITE OCCUPANCY RATES WHEN DETECTION PROBABILITIES ARE LESS THAN ONE. Ecology. 2002;83: 2248–2255. doi:10.1890/0012-9658(2002)083[2248:esorwd]2.0.co;2

17. Tingley MW, Stillman AN, Wilkerson RL, Sawyer SC, Siegel RB. Black-backed woodpecker occupancy in burned and beetle-killed forests: Disturbance agent matters. Forest Ecol Manag. 2020;455: 117694. doi:10.1016/j.foreco.2019.117694

18. Tingley MW, Ruiz-Gutiérrez V, Wilkerson RL, Howell CA, Siegel RB. Pyrodiversity promotes avian diversity over the decade following forest fire. Proc R Soc B. 2016;283: 20161703. doi:10.1098/rspb.2016.1703

19. Plummer M. JAGS: A program for analysis of Bayesian graphical models using Gibbs sampling. 2003.

20. R Core Team. R: A language and environment for statistical computing. 2019. Available: https://www.R-project.org/

21. Su Y-S, Yajima M. R2jags: A Package for Running JAGS from R. 2014. Available: http://CRAN.R-project.org/package=R2jags

22. Gelman A, Carlin JB, Stern HS, Rubin DB. Bayesian Data Analysis. Second ed. CRC/Chapman & Hall; 2004.

23. Fontaine JB, Donato DC, Robinson WD, Law BE, Kauffman JB. Bird communities following high-severity fire: Response to single and repeat fires in a mixed-evergreen forest, Oregon, USA. Forest Ecol Manag. 2009;257: 1496–1504. doi:10.1016/j.foreco.2008.12.030

24. Nappi A, Drapeau P, Saint-Germain M, Angers VA. Effect of fire severity on long-term occupancy of burned boreal conifer forests by saproxylic insects and wood-foraging birds. Int J Wildland Fire. 2010;19: 500–511. doi:10.1071/wf08109

25. Nappi A, Drapeau P. Pre-fire forest conditions and fire severity as determinants of the quality of burned forests for deadwood-dependent species: the case of the black-backed woodpecker. Can J For Res. 2011;41: 994–1003. doi:10.1139/x11-028

26. Raphael MG, Morrison ML. Decay and Dynamics of Snags in the Sierra Nevada, California. Forest Science. 1987;33: 774–783. doi:10.1093/forestscience/33.3.774

27. Lowell EC, Rapp VA, Haynes RW, Cray Caitlin. Effects of fire, insect, and pathogen damage on wood quality of dead and dying western conifers. Gen Tech Rep Pnw-gtr-816 Portland Or U S Dep Agric For Serv Pac Northwest Res Stn 73 P. 2010;816. doi:10.2737/pnw-gtr-816

28. Grayson LM, Cluck DR, Hood SM. Persistence of fire-killed conifer snags in California, USA. Fire Ecol. 2019;15: 1. doi:10.1186/s42408-018-0007-7

29. Morrison ML, Raphael MG. Modeling the Dynamics of Snags. Ecol Appl. 1993;3: 322–330. doi:10.2307/1941835

30. Dunn CJ, Bailey JD. Temporal dynamics and decay of coarse wood in early seral habitats of dry-mixed conifer forests in Oregon's Eastern Cascades. Forest Ecol Manag. 2012;276: 71–81. doi:10.1016/j.foreco.2012.03.013

31. Ritchie MW, Knapp EE, Skinner CN. Snag longevity and surface fuel accumulation following post-fire logging in a ponderosa pine dominated forest. Forest Ecol Manag. 2013;287: 113–122. doi:10.1016/j.foreco.2012.09.001

32. Landram FM, Jr WFL, Atzet T. Demography of Snags in Eastside Pine Forests of California. USDA Forest Service General Technical Report PWS-GTR-181; 2002 pp. 2–4.

33. Iknayan KJ, Tingley MW, Furnas BJ, Beissinger SR. Detecting diversity: emerging methods to estimate species diversity. 2014;29: 97–106. doi:10.1016/j.tree.2013.10.012

34. Zipkin EF, Dewan A, Royle JA. Impacts of forest fragmentation on species richness: a hierarchical approach to community modelling. J Appl Ecol. 2009;46: 815–822. doi:10.1111/j.1365-2664.2009.01664.x

35. Rota CT, Ferreira MAR, Kays RW, Forrester TD, Kalies EL, McShea WJ, et al. A multispecies occupancy model for two or more interacting species. Warton D, editor. Methods in Ecology and Evolution. 2016;7: 1164–1173. doi:10.1111/2041-210x.12587

36. Rota CT, Wikle CK, Kays RW, Forrester TD, McShea WJ, Parsons AW, et al. A two-species occupancy model accommodating simultaneous spatial and interspecific dependence. Ecology. 2016;97: 48–53. doi:10.1890/15-1193.1

37. Zipkin EF, Zylstra ER, Wright AD, Saunders SP, Finley AO, Dietze MC, et al. Addressing data integration challenges to link ecological processes across scales. Front Ecol Environ. 2021;19: 30–38. doi:10.1002/fee.2290

38. Rota CT, Rumble MA, Lehman CP, Kesler DC, Millspaugh JJ. Apparent foraging success reflects habitat quality in an irruptive species, the Black-backed Woodpecker. Condor. 2015;117: 178–191. doi:10.1650/condor-14-112.1

39. Nappi A, Drapeau P, Giroux J-F, Savard J-PL. SNAG USE BY FORAGING BLACK-BACKED WOODPECKERS (PICOIDES ARCTICUS) IN A RECENTLY BURNED EASTERN BOREAL FOREST. Auk. 2003;120: 505–511. doi:10.1642/0004-8038(2003)120[0505:subfbw]2.0.co;2

40. Tremblay JA, Ibarzabal J, Savard J-PL. Foraging ecology of black-backed woodpeckers (Picoides arcticus) in unburned eastern boreal forest stands. Can J Forest Res. 2010;40: 991–999. doi:10.1139/x10-044

41. Hutto RL, Gallo SM. THE EFFECTS OF POSTFIRE SALVAGE LOGGING ON CAVITY-NESTING BIRDS. Condor. 2006;108: 817–831. doi:10.1650/0010-5422(2006)108[817:teopsl]2.0.co;2

42. Tingley MW, Wilkerson RL, Bond ML, Howell CA, Siegel RB. Variation in home-range size of Black-backed Woodpeckers. Condor. 2014;116: 325–340. doi:10.1650/condor-13-140.1

43. Seavy NE, Burnett RD, Taille PJ. Black-backed woodpecker nest-tree preference in burned forests of the Sierra Nevada, California. Wildlife Society Bulletin. 2012;36: 722–728. doi:10.1002/wsb.210

44. Stillman AN, Siegel RB, Wilkerson RL, Johnson M, Howell CA, Tingley MW. Nest site selection and nest survival of Black-backed Woodpeckers after wildfire. Condor. 2019;121: 1–13. doi:10.1093/condor/duz039

Fig. 1

Click here to access/download;Figure;Fig1.jpg

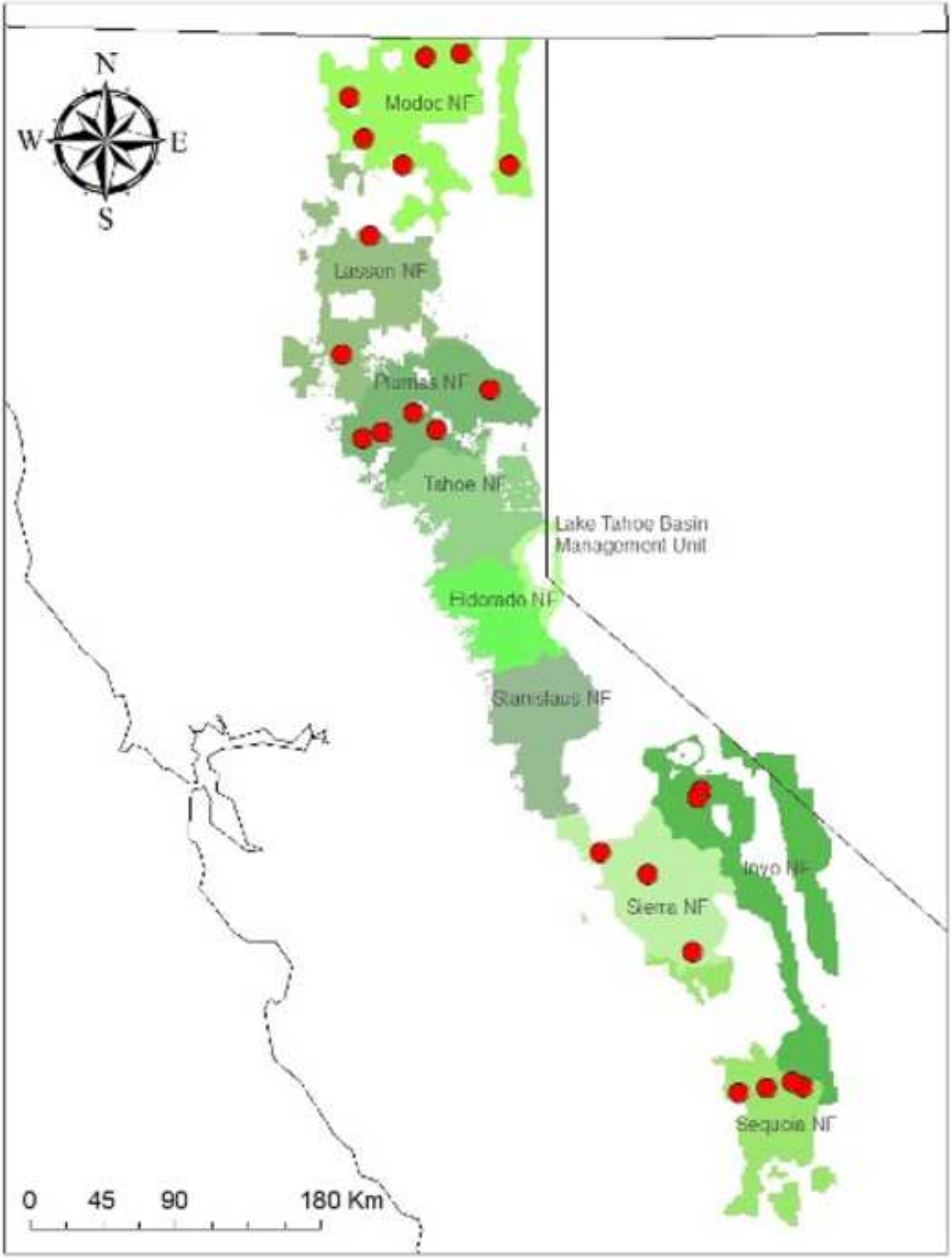

Fig. 2

Click here to
access/download;Figure;Fig2.pdf

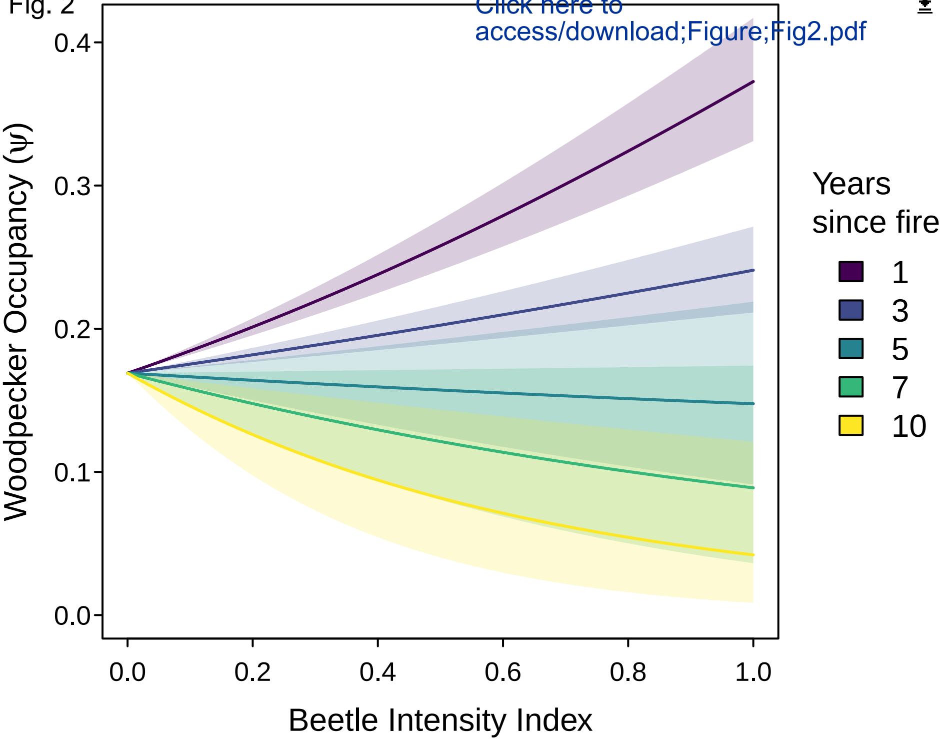

Fig. 3

Click here to access/download;Figure;Fig3.pdf

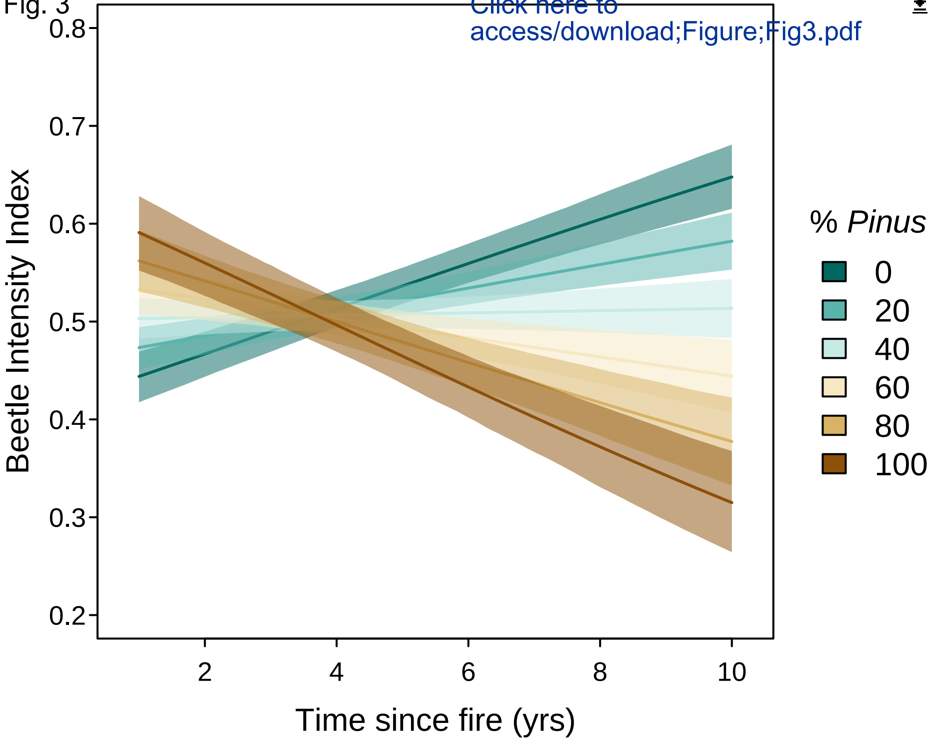

Click here to access/download
Supporting Information
jags_code.R
