# Supplementary material for: Multi-trophic occupancy modeling connects temporal dynamics of woodpeckers and beetle sign following fire
Source: PLoS One. 2023 Mar 6;18(3):e0281687. doi: 10.1371/journal.pone.0281687 (PMC9987826; doi:10.1371/journal.pone.0281687)
Supplement: Attachment — Submitted filename: Response to Reviewers.docx [file pone.0281687.s002.docx]

**Response to Reviewers**

**Summary:** We thank the editor and reviewers for their corrections and feedback on our manuscript. We have addressed every concern, specifically adding clarification and additional details to the manuscript, where necessary. Comments are addressed below with continuous numbering.

**Editor**

1. Lines 77, 141. It should be “Ray et al [13]” (italics and no dot) (it is the number, not the year). Please check if it happened with other citations (I did not find).

   Response: We have fixed this, checked, and did not find any other occurrences.
2. Lines 97. It should be “(Fig 1)”. (no dot). Please check this for all citations and captions of figures.

   Response: We have removed all dots following the abbreviation ‘Fig’.
3. Line 276. Maybe, “;” should be replaced by “)”. It is strange.

   Response: This was an error. Fixed.
4. Reference 7. Initials should not be in capitals, except in the first word, and names of regions, species, etc…Refs 16, 39 and 41 also have this problem. Please check all refs carefully again.

   Response: We have corrected and checked all of our references.
5. References 20 and 21. The underline should not be there.

   Response: We have removed embedded hyperlinks
6. Reference 32. It appears to have a problem with the second author.

   Response: Fixed
7. Reference 33. The journal is missing. Add: “Trends Ecol Evol.”

   Response: Fixed
8. Reference 40. The species name should be in italics. Please check this for all refs.

   Response: Done
9. Reference 43. Use the abbreviated name of the journal. Please check this too.

   Response: Fixed.

**Reviewer 1**

10. Manuscript "Multi-trophic occupancy modeling connects temporal dynamics of woodpeckers and beetle sign following fire" is a unique set of long-term data that sheds light on the relationship between different components of ecosystems. The manuscript is of particular interest in view of the fact that the data obtained in it can be used both in protection and in management. Statistical analysis in these ms been performed appropriately and rigorously. Manuscript written in good English. This ms certainly deserves publication in this journal. Despite this, I have a number of comments and suggestions.

    Response: We thank the reviewer for their comments on our manuscript.
11. Keywords. The article talks a lot about dead and dying trees, perhaps the authors should include this in the keywords, instead of repeating the name of the woodpecker in English and Latin?

    Response: There is no limit on keywords for this journal, so we added the following keywords: tree mortality, forest management, and dead wood.
12. L 117 - Were fluctuations taken into account in the long-term number of woodpeckers? As studies show, the food supply is not the only factor affecting the number and occurrence of woodpeckers. The number of beetles can be very high, and there can be no woodpeckers due to low numbers, for example, due to poor breeding or a harsh winter that led to a strong decrease in numbers.

    Response: Previous work in this system (e.g., Tingley et al. 2018 *Journal of Animal Ecology*) has shown that abundance of Black-backed Woodpeckers are particularly stable from year to year within fires, with very little inter-annual fluctuations. Populations within fires do decline predictably over longer time spans, as the quality of habitat (e.g., food availability within dead trees) declines over 10–20 years. Our model explicitly accounts for this long-term temporal process (line 211). Any year-to-year variance in occurrence that arises from stochastic processes – which we expect to be small based on previous work on this species – would remain in the model error term.
13. L 187-189. Sorry, but are you sure this is the best way to convert categorical variables to scale variables?

    Response: This is a good question and there is no ‘perfect’ solution. The variable, as collected, is technically ordinal: snag counts of bins ≤5, 6-15, 16-30, 31-50, 51-100, >100. The best way to analyze this type of variable depends on one’s hypothesized relationship. In this case, we expect the relationship to be monotonic and saturating. In other words, we expect that more snags are always better, but that the effect of the 101^st^ snag is much less than the 2^nd^ snag. If we treated the variable as categorial in our model, then the ordinal relationship would be lost – each group would be treated as independent, which would both sacrifice degrees of freedom and miss out on the benefit of our a priori belief that the relationship is monotonic. We could also potentially treat the data as simply ranked (i.e., in a non-parametric analysis), where the bins simply become integers 1 through 6. While this maintains the monotonic nature of the data, the covariate itself becomes harder to interpret, because the slope is based on the addition of a rank (which isn’t meaningful). Ultimately, we chose the method we did because even though the data are binned, the bins are numerical (i.e., based off of real numbers, not subjective categories like “a few” or “many”) and we believe those numbers have meanings. While we chose to represent the bins by the minimum of each bin (i.e., the quantities 1, 6, 16, 31, 51, and 101), we could have chosen other benchmarks (e.g., midpoints), but that is a minor subjective choice and would produce qualitatively identical results. We have edited a sentence in our Methods to clarify that these numbers produce a ‘relative index’ of snag density.
14. L 205-207. This is very interesting and novel, but I have some doubts that it is "continuous"

    Response: We accept that in the real world, variables that we measure are not always purely continuous, and that ecologists must make choices about how we quantify phenomenon and we must be careful that what we measure is connected to reality. In this particular case, our model is estimating that there is some true, un-measurable (by current abilities) but still biologically real variable which is the amount of wood-boring beetle activity that has occurred. While we could likely never directly measure such a variable, every measurement we do take (e.g., bark surveys) is an indicator of what that true variable is. Our model uses those multiple bark surveys as indicators and then estimates what this true underlying variable is. Since this ‘latent’ variable has no units and serves as a theoretical index, it is simplest and most mathematically straightforward to parameterize it as a continuous variable. We have edited this line to now specify that the variable is defined as continuous for mathematical reasons.

**Reviewer 2**

15. One of my main concerns is that survey stations are used as the independent unit of analysis. Additional information is required to justify this decision.
    For example, what was the average distance between survey stations (and standard deviation)? Did this distance vary based on the size of the fire perimeter?
    As the manuscript is written, this critical issue does not receive sufficient discussion.

    Response: We apologize for not making this very clear in our previous submission, we have addressed this in two ways. First, we have added a paragraph to the Methods section to add additional clarity on the topic. That paragraph (beginning line 138) reads:

    "The six survey points per transect comprise the basic analytical unit for black-backed woodpecker occurrence in our analysis. The mean minimum distance between these points is 472 m. Point count standards generally recommend a minimum inter-point distance of 250 m in order to minimize double-counting, although we acknowledge that the use of broadcast surveys has the potential to attract birds from surrounding areas. However, telemetry work on black-backed woodpeckers in this system shows that independent individuals travel at a velocity of approximately 0.75 km per hour, and a max rate of <1.2 km per hour [16]. With surveys lasting a median of 7 minutes per point and points separated by approximately 500 m, birds would have to travel approximately twice their maximum normal velocity to keep up with survey broadcasts. Regardless, our analysis of woodpecker occurrence can equally be considered an analysis of "use" should there be any risk of non-independence across survey points [14]."

    Secondly, we have re-run our full analysis, adding a random effect for fire into the occupancy analysis to reflect the multiple sampling of points within fires. This random effect effectively creates a different intercept for every fire, thus accounting for variation that may be unique to individual fires which previously was explained by other variables. Our results remain largely unchanged after the inclusion of this factor.
16. L 20: Using hypothesis as a verb is never a sound idea.

    Response: We have re-written this sentence to avoid the verb “hypothesize”.
17. L 59: This? Please avoid ambiguous references.

    Response: We have corrected the ambiguous reference of “this” in the sentence referenced, and have searched the manuscript and corrected 2 other ambiguous references.
18. L 71: What types of similarities?

    Response: We have clarified the sentence to be more precise about the specific knowledge gap.
19. L 75: Are beetle populations the only factor influencing distribution of woodpeckers? What about adequate nesting substrates? Competition? Predation?

    Response: We have provided additional caveats on this hypothetical use, mentioning competition and predation specifically. However, based on our previous work on this species, we don’t find strong evidence for nesting limitation (i.e., they select for nesting stands that largely mirror food availability and suitable nesting sites exist in many areas where the species is absent) or competition (i.e., post-fire areas have very high woodpecker species richness and we have only very rarely observed interactions that indicate competitive displacement). While predation (particularly of newly fledged woodpeckers) can be high, there are no specialist predators on this species that we are aware of.
20. L 82: Writing in this fashion will confuse readers. What relationship has been stated about dominant tree species and their association with BBWO? If no mechanistic relationship has been identified, what is the basis of the hypothesis?

    Response: We have re-written the sentence as follows to clarify what is known versus unknown:

    "Black-backed woodpeckers in California have previously been associated primarily with trees of the genus *Pinus* within mixed conifer forests [14], but a mechanistic relationship tying woodpeckers to particular tree species as a function of time since fire has never been clarified."
21. L 109: What is the average territory size of a BBWO? Given these surveys involved using a caller, it is possible that BBWO moved from one "site" (as the survey stations are treated) to another "site" during the surveys? This event seems possible, and later an assumption at the survey level is described. I think this situation has to be discussed in greater detail, and with a clear description of what the sample units and experimental units are in the study.

    Response: Please see response to comment #15 where we respond in detail to this concern.
22. L 143: Within an individual fire, what is the basis for treating the survey units as independent? Shouldn't the beetle populations be responding in the same fashion at the survey site scale within a fire perimeter?

    Response: Please see response #15. We have now added a random effect of fire to our model, which accounts for any potential pseudoreplication caused by sampling multiple times within fires. We additionally note that beetle abundance and sign shows large amounts of heterogeneity within fires – even among trees within survey points – as a result of variation in tree density, tree species composition, aspect and slope, and burn severity.
23. L 175: What is a hypothesized covariate?

    Response: We have rewritten the sentence as, “The probabilities of woodpecker detection and occurrence are both modeled as logit-linear functions of covariates chosen *a priori*.”
24. L 236: The predicted values for the second result vary from 0.05-~0.35. So, beetle index predicts BBWO occurrence when occurrence varies from unreliably low to moderately low. This distinction requires more discussion in the manuscript.

    Response: For mobile species, occupancy estimates are always relative to the space and time in which surveys are conducted, which is a fundamental issue when comparing absolute occupancy estimates across studies. In this case, our estimate of occupancy is best defined as the probability of use of an approximately 120-m radius circle by a black-backed woodpecker during a survey period (which lasts a median of 7 minutes). We now explicitly define this on Line 188. Thus, in this context, an occupancy of 0.35 is not ‘moderately low’ but actually quite high.
25. L 263: Why are some estimates described as associations and some as effects?

    Response: Our mistake, they should all be defined as ‘associations’. We have corrected this.
26. L 268 (Table 2 legend): An estimate can be very small and very precise.

    Response: We intended to suggest that non-overlapping credible intervals imply estimates for which we know with greater certainty where they lie (i.e., what would normally be problematically called ‘significance’). We have rephrased the table legend as:
    “Bayesian credible intervals that do not cross zero suggest relationships for which we have strong evidence and are highlighted in bold.” We have also checked and corrected for any problematic language in the presentation of results, particularly making sure we describe findings as strong or weak 'evidence for an association' not strong or weak 'associations' (as the former implies certainty about the existence of an association and latter suggests the magnitude of the association).
27. L 269 (Table 2): Where is the estimate for fire age? If you are including an interaction between beetle sign and fire age in the model, but covariates have to be present individually.

    Response: Thank you for this query as we had not adequately explained it previously. Our model assumes that all non-random temporal changes in occupancy manifest solely through the temporal changes in habitat quality, as indicated by beetle sign. In other words, there is no other mechanistic hypothesis by which we imagine Black-backed Woodpecker occupancy to decline over time (see also response #19), thus there is no need for an independent effect of time-since-fire on occupancy. We now explain this reasoning through a new paragraph added to the Methods (lines 225–231).
28. L 350: So, rather than survey potential habitat for BBWO, the suggestion is to survey for beetles?

    Response: We intend our takeaway message to be a better understanding of the complexity of the temporal connection between the multiple trophic levels present in post-fire forests, each of which is imperfectly surveyed. It is not enough for managers to survey woodpeckers at just one point in time and assume their distribution is fixed, it is critical to understand where they go over time and how resource use shifts in these dynamic post-fire forests. In order to best understand these temporal dynamics, we need to understand the linkages across trophic levels.
29. L 351: “The nuanced but strong relationship…”???

    Response: We have edited it to be: “The temporally-variable but strong relationship…”
30. L 353: I am not certain this study demonstrates that BBWO depend on woodboring beetle abundance exclusively especially since occurrence at the survey site scale is hardly a way to monitor population size or dynamics.

    Response: We have re-written the sentence to increase the precision of our claims.
